# Familial Takayasu arteritis - a pediatric case and a review of the literature

**DOI:** 10.1186/1546-0096-9-6

**Published:** 2011-02-02

**Authors:** Kimberly A Morishita, Karen Rosendahl, Paul A Brogan

**Affiliations:** 1 Division of Rheumatology, Department of Pediatrics, British Columbia's Children's Hospital, University of British Columbia, Vancouver, BC, Canada; 2 Department of Radiology, Institute of Child Health and Great Ormond St Hospital for Children, London, UK; 3 Department of Rheumatology, Institute of Child Health and Great Ormond St Hospital for Children, London, UK

## Abstract

Takayasu arteritis (TA) is a rare chronic inflammatory disease of the aorta and its major branches. It is seen predominantly in females during the second and third decades of life, although it can occur in childhood. The aetiology of TA remains unknown. To date, familial cases of TA have been considered rare; however, a review of the literature suggests that cases are accumulating. We report a case of two sisters affected by severe TA, and review other reported familial cases.

## Background

Takayasu arteritis (TA) is a chronic, idiopathic large vessel vasculitis. It primarily involves the aorta and its main branches. Although the aetiology of TA has been extensively investigated, it remains unknown. The disease is more common in females and is seen predominantly in Asian countries although it has a worldwide distribution. Despite several reported associations with HLA alleles, results are heterogeneous and differ across populations. A review of the literature suggests that familial TA may not be as rare as once thought and this finding may have both aetiological and clinical implications for family screening. The following case describes a 4-year old girl with severe TA whose older sister died of the same disease.

## Case

Our female patient was born to non-consanguineous Italian parents in 1990 after an uneventful pregnancy. Both parents were healthy and the family history was unremarkable except for the following history in her sibling. Her older sister had been diagnosed with TA in Belgium at the age of 10 years. Only limited information was available to us regarding this sibling: she had a history of severe hypertension and persistently elevated acute phase markers (high erythrocyte sedimentation rate, ESR; and high C-reactive protein, CRP) as well as involvement of her cerebral, abdominal, and renal arteries. At the age of 15 years this sibling died of a massive cerebral haemorrhage. No autopsy was performed; therefore, the cause of the haemorrhage was never identified. Our patient was transferred to Great Ormond Street Hospital (GOSH), London in 2006 after a severe and resistant course. She had been diagnosed with TA at the age of 4 years after presenting with hypertension and persistently raised inflammatory markers (ESR 50-105 mm/hour; CRP 40-95 mg/L). Catheter arteriography at the time of diagnosis revealed stenotic abnormalities of the superior mesenteric, renal and internal carotid arteries. Her initial management included prednisone, nifedipine, and labetolol. Over the next several years she developed impaired renal function with glomerular filtration rates between 50-60 ml/min/1.73 m^2 ^and had ongoing hypertension despite addition of enalapril and commencement on azathioprine. In 2002, at the age of 12 years she underwent bilateral balloon dilatation of her renal arteries. In 2004 she had three intracranial haemorrhages from a basilar artery aneurysm, which was subsequently coiled. Between 2003 and 2006 she developed recurrent gastrointestinal complaints of abdominal pain, diarrhoea and weight loss due to superior mesenteric artery (SMA) stenosis and secondary intestinal ischemia. She required balloon dilatation of the SMA on four separate occasions. During the same time period she was diagnosed with episodic atrial fibrillation requiring treatment with flecainide. Her echocardiogram showed bilateral ventricular hypertrophy, mild aortic regurgitation, and mild stenosis of the distal main pulmonary artery. The atrial fibrillation and cardiac changes were felt to be secondary to longstanding hypertension in the context of large vessel vasculitis. Acetylsalicylic acid (ASA) or other antiplatelet agent was not initiated due to the patient's previous history of intracranial bleeds, precarious vasculature, and persisting hypertension.

**Figure 1:**
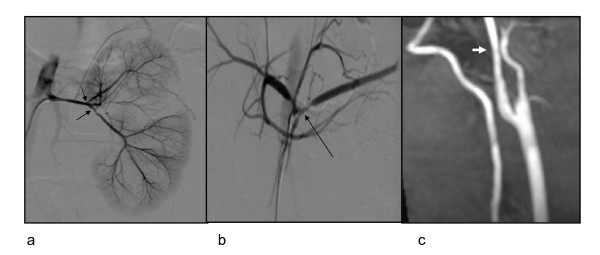
**Angiogram from 14.09.2006, showing a) narrowed renal arteries, and severe stenoses of their main intrarenal braches (arrows), b) celiac trunk and its main branches are stenosed at its trifurcation (arrow) c) magnetic resonance angiography from 10.11.2006 showing smooth narrowing of the proximal right internal carotid artery**.

At 16 years of age (in 2006) she was transferred to GOSH for a second opinion. She had severe hypertension (systolic blood pressure on average 230 mmHg), severe failure to thrive (weight 21 kg; height 129 cm, both significantly below the 3^rd ^percentile; body mass index 12.6 kg/m^2^), chronic renal failure (glomerular filtration rate 52 ml/min/1.73 m^2^), chronic diarrhoea and intestinal malabsorption from ischaemia, and osteoporosis with vertebral compression fractures. She largely used a wheelchair and could only walk approximately 50 metres, mobility mainly limited by general weakness and musculoskeletal debilitation. Physical examination revealed a pale, pre-pubertal girl. She had loud bilateral carotid artery bruits, a left renal artery bruit and otherwise normal peripheral pulses. The ESR was persistently elevated (70 mm/hour) and she had mild anaemia of chronic disease (haemoglobin 9.8 g/dL). Catheter digital subtraction arteriography (DSA) and magnetic resonance angiography (MRA) findings revealed widespread arterial pathology, which combined with the ongoing chronic inflammation (in the absence of other identified causes) was consistent with TA (Figure 1). Between 2006 and 2007 the patient's clinical status remained stable on moderate doses of oral prednisone (0.5 mg/kg/day), azathioprine 2 mg/kg/day, and multiple antihypertensive medications, although the ESR remained persistently elevated (ranging from 30-80 mm/hour over several measurements months apart). Pamidronate infusions for osteoporosis were given monthly. In 2007, she developed pain and stiffness in both hips and an MRI confirmed the presence of bilateral hip joint effusions with thickened synovium as well as significant synovial enhancement. Due to presumed hip synovitis azathioprine was switched to subcutaneous methotrexate (15 mg/m^2 ^weekly). Despite attempts to lower her baseline prednisone dose, she could not be weaned below 0.5 mg/kg/day. The patient initially declined anti-tumor necrosis factor (TNF) alpha therapy (because of numerous concerns including needle phobia), although at the age of 17 years adalimumab 20 mg subcutaneous fortnightly was added to her treatment in an attempt to spare her baseline corticosteroid dose [1]. This resulted in normalisation of the ESR within receiving 2 doses after which the patient refused to continue with this treatment because of pain associated with the injections. At the time of writing she has been transitioned to adult care and remains on prednisone and methotrexate, in addition to multiple anti-hypertensive agents. Ongoing palliative care of symptoms is now the primary therapeutic goal.

## Discussion

Individual case reports of familial TA consistently refer to the rarity of this condition. We reviewed the literature and found 30 other reports, the majority of which were from Japan (Table 1) [2-16]. An extensive amount of research has focussed on HLA associations. Although some consistent HLA associations have been identified with HLA- A10, B5, Bw52, DR2, DR4, B21 and B22 [17-22], many of these remain unconfirmed and variable across different ethnic groups. A recent report of a multiplex family with TA raised the question of whether there could be an autosomal recessive form of the disease [5]. We concur with the authors who propose the use of homozygosity mapping in these cases. Homozygosity mapping may enable us to identify candidate genes involved in the pathogenesis of TA, particularly in consanguineous families [23], and could ultimately provide insight into non-familial TA.

**Table 1:** Summary of familial cases of Takayasu arteritis

Case	Affected family members	Country	Possible genetic associations	Reference
1	Monozygotic twins	Japan	HLA A11, Bw40, Bw52	[4]
2		Japan	HLA A9, A10	[3]
3		Japan	HLA Aw31-Bw52	*
4	Sisters	Japan	-	[13]
5		Europe	-	[6]
6		Japan	-	*
7		Japan	HLA Aw24-Bw52	*
8		Japan	HLA Aw24-Bw52	*
9		Japan	HLA Aw24-Bw40	*
10		Japan	HLA Aw24-Bw52-DR2-MT1	*
11		India	-	[7]
12		Europe	-	[11]
13		Taiwan	HLA DR4	[8]
14		India	HLA B5	[9]
15		Europe	-	present case
16	Brothers	Europe	-	[14]
17		Japan	HLA A9, DRw4	[15]
18		Japan	HLA DR2, DR4	[12]
19	Brother and sister	Japan	HLA Aw24-Bw52	*
20		Japan	HLA Aw24-Bw52-Dw12	*
21	Mother and daughter	Japan	-	^†^
22		Japan	HLA Bw52-DR2	*
23		Japan	-	^†^
24		Japan	HLA Aw19-Bw52-Dw12	*
25		Japan	-	[10]
26	Aunt and niece	Japan	-	*
27		Japan	-	*
28		Japan	HLA Aw24-Bw52-DR2-MTI	*
29		Korea	-	[16]
30	Cousins	Japan	HLA Bw52	*
31	Multiple siblings	Pakistan	-	[5]

* Cited in reference [2]

^† ^Cited in reference [3]

-- Genetic testing not performed or no association reported

Our literature search revealed that a large proportion of familial cases were reported over 15 years ago. The reason for this is unclear. The availability and quality of imaging modalities used in the past may have affected diagnostic accuracy; however, for those cases in which clinical details were provided, many cases had angiographic findings typical of TA suggesting that the diagnoses were reliable (3, 8, 9, 12, 15). Most of the older studies originated in Japan, and it is possible that even if new cases are occurring, they are simply not being reported unless they are unique in some way.

Our review of familial cases also revealed that many affected siblings were only diagnosed after they presented with late signs and symptoms of disease, without having previously been evaluated for absent pulses, four-limb blood pressure inequality, or hypertension [8,9,15]. This was especially true for non-twin cases. Tsai and colleagues report a case of two teenage sisters in which there was a significant delay in diagnosis of the second sister in part due to failure to palpate pulses and obtain 4-limb blood pressures at the time of initial evaluation [8]. An asymmetric radial pulse was noted incidentally on follow-up examination after a two month history of fever of unknown origin and vague left arm pain, even though the patient's older sister had previously been diagnosed with TA. Makino and colleagues report a case of two brothers aged 17 and 14 years, where the younger brother was diagnosed 4 years after the older brother when he presented with neck pain and general malaise. Physical examination revealed blood pressures of 144/60 and 160/54 mmHg in the right and left arms, respectively. Arteriography showed a grossly dilated aortic arch and bilateral carotid artery narrowing [15]. Other cases are similarly described with delayed diagnosis of familial TA with considerable late disease related damage and large vessel injury [12].

The diagnosis of TA is not always straightforward and is based on angiographic abnormalities of the aorta or its major branches along with other clinical or laboratory features to support the diagnosis of a systemic vasculitis. The differential diagnosis for TA is broad and is summarized in Table 2. Although the pattern of vessel involvement of both siblings reported in this case was somewhat atypical, the most likely diagnosis was felt to be TA given the constellation of clinical, laboratory, and radiologic findings demonstrating arterial lesions affecting major branches of the aorta. It is entirely possible, however, that these two siblings and perhaps some of the cases reported in the literature actually represent an as yet undefined and unusual genetic inflammatory vasculopathy mimicking TA. We suggest that detailed genetic study of such informative families using new genetic technologies such as homozygosity mapping followed by second generation resequencing of areas of homozygosity could reveal novel genetic causes of unusual familial large vessel vasculitis of this nature.

**Table 2:** Differential diagnosis for Takayasu arteritis

Infections	Septicemia or endocarditis (mycotic aneurysms)
	Tuberculosis
	Syphilis
	Human immunodeficiency virus
	Borelliosis
	Brucellosis
Inflammatory vasculitides	Giant cell arteritis (adults)
	Kawasaki disease
	Polyarteritis nodosa
	Wegener's granulomatosis

Autoimmune conditions	Systemic lupus erythematosus
	Rheumatic fever
	Sarcoidosis

Non-inflammatory vasculopathies	Fibromuscular dysplasia
	William's syndrome
	Congenital coarctation of the aorta
	Congenital mid-aortic syndrome
	Ehlers-Danlos type IV
	Marfan syndrome
	Neurofibromatosis type I

Other	Post radiation therapy

## Conclusion

Familial TA is an important clinical entity, which may not be as rare as we once thought. This case and review provides further support for the role of as yet undefined genetic factors in the development of TA, and emphasizes the need for studying such informative familial cases to look for candidate genes. In addition, physicians should maintain a high index of suspicion for the possibility of TA in the siblings or other family members of affected patients. Screening using four-limb blood pressure measurements and pulse checks should be considered in these cases, progressing to non-invasive imaging such as ultrasound followed by MRA if abnormalities are detected. Such relatively non-invasive screening may prevent delays in diagnosis and minimize morbidity of other familial cases.

## Consent

Written consent was obtained from the patient's mother for this publication.

## Competing interests

The authors declare that they have no competing interests.

## Authors' contributions

KM and PB wrote the initial manuscript draft. KR performed the analysis and interpretation of radiological data. All authors critically reviewed and revised drafts. All authors read and approved the final manuscript.
